# Enhancing the odds of adopting e-learning or community-focused experiential learning as a teaching practice amongst university faculty

**DOI:** 10.1016/j.heliyon.2021.e06704

**Published:** 2021-04-16

**Authors:** Robindra Sidhu, William H. Gage

**Affiliations:** a Office of the Associate Vice-President, Teaching & Learning, York University, Canada; b School of Kinesiology and Health Science, York University, Canada

**Keywords:** Innovation adoption, Organizational change, Experiential learning, e-learning, Faculty motivation, Self-efficacy, Institutionalization

## Abstract

Identifying the optimal approach for motivating faculty to adopt teaching innovation is important, given that broad-scale initiatives can utilize an inordinate amount of time and resources. Using a quantitative approach, we evaluate policy actions that are most strongly associated with the adoption of either e-learning or community-focused experiential learning, over a five-year period in a single institution. Comparisons between adopters and non-adopters affirm the relevance of previously documented facilitators and barriers. However, a logistic regression analysis demonstrates that actions that promote a supportive institutional culture (such as, an institutional plan, committee involvement, professional development and logistical support) as well as faculty perceptions and beliefs (i.e., “using new methods is not risky for student learning”; confidence and self-efficacy with respect to implementation), is strongly associated with the adoption of either e-learning (*n* = 118) or community-focused experiential learning (*n* = 97). In contrast, funding and professional dimensions (i.e., workload, historical precedence, and the institutional promotion of the innovation with respect to academic freedom) is weakly associated with adoption. The results not only provide a fine-grained analysis of current assumptions regarding the necessary conditions for implementing organizational change in the university context, but also suggest an approach that reinforces and sustains the adoption of teaching innovation over the long term. Theoretical and practical implications are discussed in reference to models of organizational change, faculty motivation and approaches to institutionalizing teaching innovation.

## 1 Introduction

Motivating university faculty to adopt teaching innovation is of enduring interest to both researchers and decision-makers (Averill and Major, 2020; Genné-Bacon et al., 2020; Terantino, 2020). Indeed, broad-scale initiatives to enhance student learning can utilize an inordinate amount of time, effort, and resources. University leadership must consider the needs and expectations of students and faculty, and ultimately of government who fund operations. Within the context of the urban university, students have a diverse range of socioeconomic, educational, and professional aspirations. Many students commute, work-part-time and perceive that a degree offers enhanced employability (Donald et al., 2019; García-Aracil et al., 2018; Kasler et al., 2017; Lock and Kelly, 2020) and improved employment odds (Becker, 1993; Belfield et al., 2018; Berger et al., 2009; Donald et al., 2018; Kasler et al., 2017). During the implementation of teaching innovation, faculty expect that leadership will provide support and uphold academic traditions and values that imbue the working lives of faculty members, such as excellence in teaching and research, academic freedom, collegial governance, and autonomy (Altbach, 2001; Austin, 1990; Davies, 2015; Karran, 2009). At the same time, leadership is expected to satisfy external demands by government such as an increased focus on accountability metrics such as student retention, persistence/degree progression, graduation rates and employment rates, which in turn are increasingly linked to performance-based funding (e.g., Ziskin et al., 2014), despite unintended consequences (Gándara and Rutherford, 2018).

One possibility is to foster the conditions that enable and incentivize faculty members so that they are more likely to adopt innovative teaching approaches that engage student learning. Faculty-adoption of teaching innovation is one way of meeting student learning needs, assuming they are demonstrated to be effective. As such, the ways in which faculty-adoption of two teaching approaches may be enhanced are examined separately here: Specifically, e-learning (i.e., blended and fully online) and community-focused experiential learning (i.e., service learning, community-based learning & community-based research). Although we do not directly evaluate the merits or outcomes of these teaching approaches, we do however, seek to determine the optimal conditions for their broad-scale adoption.

Both e-learning and community-focused experiential learning can potentially address some learning needs of university students in engaging and inspired ways (e.g., Almarghani and Mijatovic, 2017; Biggs and Tang, 2011; Groccia, 2018; Redmond et al., 2018). For students who work full or part-time, or must commute long distances, e-learning offers the possibility of flexible, asynchronous, and remote learning. E-learning also has the potential for innovative ways of learner engagement, for example in the case of the blended or flipped classroom format. Indeed, a growing body of research suggests that blended or flipped courses can be as effective and engaging as traditional courses (Balaban et al., 2016; Casasola et al., 2017; Giannousi et al., 2014; González-Gómez et al., 2016; Halasa et al., 2020; Mattis, 2015; Sahin et al., 2015; Swart and Wuensch, 2016; Viljoen et al., 2020).

Community-focused experiential learning is appealing to students because it enables them to work with organizations that they could not otherwise. Students can gain practical experience and a deeper appreciation of the community and subject matter in context (Alsina Naudi, 2020; Biss Keller, 2019; Cowart, 2010; George-Paschal, 2019). Additionally, this approach enables students to learn about team dynamics when working with others (Ives-Dewey, 2009; Sanft and Ziegler-Graham, 2018), civic engagement (Hellwege, 2019; Manning and Hemer, 2019; McGowin and Teed, 2019; Prentice, 2007; Reddick et al., 2018; Yee, 2020), multicultural competence, awareness of inequality, and commitment to social justice (Einfeld and Collins, 2008; Finucane et al., 2018; Lee and Kelley-Petersen, 2018; Levesque-Bristol et al., 2010; Li et al., 2019; Unfried and Canner, 2019; Yee, 2020). Such experiences afford students the opportunity to reflect upon their personal, educational, and professional interests (i.e., self-discovery, personal fit, and self-actualization), and enables them to consider future educational and/or career goals, which can serve as an important step in transitioning into the next phase of their lives (Gault et al., 2000). From a pedagogical perspective, community-focused experiential learning promotes active learning (Brand et al., 2019; Chan et al., 2019; Dealey, 2020; Hosman and Jacobs, 2018; Natadjaja, 2019) and provides a way in which material can become more relevant, meaningful and clearly understood (Paul and Mukhopadhyay, 2005). Indeed there is evidence that such approaches are not only positively associated with deeper and more engaged approaches to learning (Archiopoli and Murray, 2019; Coker et al., 2017; Gomez-Lanier, 2016; Ives-Dewey, 2009; Trigwell et al., 1999; Trigwell and Prosser, 1991), but are positively associated with government accountability indices such as improved student retention (Gallini and Moely, 2003; Mungo, 2017; Rochford, 2013; Song, 2017) and increased persistence in undergraduates (Reed et al., 2015), and persistence within low income students and first generation students (Yeh, 2010), higher GPA (Mungo, 2017; Rochford, 2013; Song, 2017; Vogelgesang and Astin, 2000) and increased probability of degree attainment (Lockeman and Pelco, 2013; Mungo, 2017; Song, 2017).

Regardless of the potential benefits of these teaching approaches, the mere presence of an institutional commitment and provision of faculty support does not necessarily result in faculty adoption. This is not surprising given the variety of competing interests and pressures that impinge upon decisions at the institution or faculty level (Frambach and Schillewaert, 2002). Organizational-change researchers emphasize the importance of the individual within the organization as key to bringing about desired change. For example, Weiner et al. (2008) describe organizational change as the coordinated action by many individuals. Choi and Ruona (2011) view individuals as being active agents in processing and bringing about change. In other words, while the notion of innovation adoption may be conceived and facilitated at the institutional level, the actual locus of change and its implementation occurs at the level of the individual (Haque et al., 2016; Thompson, 2019). Given variation in faculty adoption, a fundamental question is why some faculty are more likely to adopt teaching innovation whereas others, less likely.

## 2 Focus of the present study

The present study is a retrospective examination of the adoption of e-learning or community-focused experiential learning by individual faculty members within a single publicly funded urban university, following a five-year sustained period of institutional promotion and support for these teaching approaches. As such, the present study seeks to determine the relative importance of institutional policies and practices, many of which were implemented in the manner that are consistent with models of organizational change and can also be traced to the literature on the barriers and facilitators that influence faculty adoption of e-learning and community-focused experiential learning (as described below). Using a logistic regression approach to predict faculty adoption of a given teaching practice, the current study evaluates the relative importance of an institutional culture that promotes readiness for organizational change (i.e., having an institutional plan, faculty committee involvement) and capacity building (i.e., faculty professional development, technical/logistical support), extrinsic rewards (e.g., funding), as well as a consideration of faculty members’ perceptions and beliefs (e.g., self-efficacy; perceived effectiveness of the teaching approach) and other professional dimensions (e.g., historical precedence in the academic field, academic freedom, time commitment). In doing so, one can determine which policy options are most strongly associated with the adoption of such teaching practices. Such knowledge not only provides a fine-grained analysis of previously understood assumptions regarding the necessary conditions for implementing organizational change in the university context, but also provides some insight and direction in terms of what is likely to reinforce and sustain the adoption of teaching innovation over the long term.

The present study will answer the following questions:

• How do adopters compare with non-adopters, on variables highlighted in the extant literature?
• What variables are uniquely associated with faculty adoption of either e-learning or experiential learning, after accounting for all other variables?
• In reference to faculty adoption what is the relative importance of the categories of variables being examined? (i.e., demographics, funding, institutional culture, faculty perceptions/beliefs, and professional dimensions)

## 3 Models of organizational change: from individual adoption to institutionalization

One characteristic of the successful adoption of a teaching innovation is that its value is broadly recognized by faculty, and it becomes an integral part of teaching practice. In other words, the teaching practice becomes institutionalized, and is no longer viewed as novel or innovative (Colbeck, 2002; Holland, 2009; Pina, 2008). Models of organizational change offer a prototypical and somewhat idealized strategy in terms of how leadership can introduce and implement new initiatives in a sustainable fashion. Although there is some variation across models (e.g., Armenakis et al., 1993; Elrod and Kezar, 2017; Kotter, 1995; Van der Voet, 2016) they can be roughly characterized as having three overlapping phases: organizational readiness/pre-implementation, adoption/implementation and, institutionalization.

### 3.1 Organizational readiness/pre-implementation

During the first phase the leadership seeks to prepare members for the intended change with a view to explain why the change is necessary and mitigate any resistance that may be present (Armenakis et al., 1993; Armenakis and Harris, 2002; Haque et al., 2016). The change message conveys a sense of (i) discrepancy -- that the organization is not where it should be; (ii) efficacy – that individuals can succeed in implementing the change; (iii) appropriateness – that successfully argues the intended course of action is correct; (iv) principal support – that individuals will be supported during implementation (Cole et al., 2006); and (iv) personal valence -- that individuals’ self-interest is not at stake because of the change (Armenakis and Harris, 2002; Elias, 2009). These elements are part of an influence strategy that includes (i) persuasive communication -- through various modes (e.g., speeches & public fora, planning documents or discussion papers Cole et al., 2006; Haque et al., 2016; Van der Voet, 2016) (ii) active participation of individuals -- through various working groups or guiding coalitions to enact decisions that affect them (Armenakis et al., 1993; Choi and Ruona, 2011; Fernandez & Rainey, 2006) so that individuals are empowered, and take part in creating the plans for the intended change; and (iii) through the management of information – for example, by showcasing examples of successful innovation adoption at other institutions, using multiple sources for delivering a consistent message, and utilizing external speakers and experts (e.g., Armenakis et al., 1993). The influence strategy is viewed as an “unfreezing” step, originally coined by Lewin (1947), and is designed to alter the perceptions of individuals so that they believe the change will be necessary and successful. Indeed, authors who emphasize the importance of this phase (e.g., Armenakis et al., 1993; Armenakis and Harris, 2002; Choi and Ruona, 2011) believe that any change message should be done with a high degree of urgency, and frequency (Kotter, 1995). From the perspective of the individual faculty member, this period is marked by an initial awareness of the innovation, and as such, they may be engaged in information-gathering so that they may decide whether to make the commitment for innovation adoption (Busick and Inos, 1994). l.

### 3.2 Adoption/implementation

During the second phase some organizational members begin to incorporate the innovation into their own practices. From the perspective of the individual, by focusing their energy on the task of implementation, the process itself may raise concerns for the adopters in terms of how best they can make the change, navigate steps that are required, as well as how it impinges on their other responsibilities (Busick and Inos 1994). Despite advanced planning, unanticipated issues may arise. For this reason, the adoption/implementation phase is seen to be more difficult than the pre-implementation phase, owing to its complexity and the inherent degree of unpredictability (Brunsson, 2009 cited in Van der Voet, 2016). To mitigate some of these concerns, the organization may be continually developing, refining and evaluating support resources (e.g., administrative/technical, and/or professional development) that will assist current and future members in the process of innovation adoption. In addition, some authors have argued for funding and the allocation of protected time, so that adopters are able to manage the implementation amongst their competing responsibilities (Busick and Inos, 1994; Cook et al., 2009; Demb and Wade, 2012; Glass et al., 2011; Holland, 1999; McKeogh & Fox, 2009; Lenton et al., 2014; Ward, 1998). To increase the number of adopters during the second phase, the organizational leadership may continue to employ the aforementioned influence strategies. During this phase, some faculty members may choose not to adopt, or temporarily adopt and then revert to previous ways of doing things. As such, researchers have pointed out the danger of the leadership declaring victory too early, following a period of initial enthusiasm (Busick & Inos, 1994). Adopters may come to realize that change is too difficult, ineffectual (as evidenced by poor outcomes, possibly due to a suboptimal implementation), or too costly on a personal level (Lenton et al., 2014). This can result in the phenomena of an implementation dip, whereby the number of adopters no longer increases, or actually decreases. To mitigate the implementation dip, Busick and Inos (1994) have suggested that the leadership endeavours to sustain, reinvigorate, and renew its focus by conveying the underlying reasons for its change vision, both on an individual and institutional level.

### 3.3 Institutionalization

Finally, during the third phase the organizational members come to accept the value of the innovation and see it as a normal part of operations. Following a period of implementation, faculty members may turn their attention to refining and improving upon their practices. From an individual perspective, this period is characterized as being one of refocus and renewal (Busick and Inos, 1994). In doing so, faculty may turn to other colleagues who are engaging in similar activity (e.g., a community of practice). From a broader organizational perspective, institutionalization can be manifested in three dimensions: At the level of (i) rules and regulations – so that the institution can define what and how such forms of teaching can be recognized and legitimized, (ii) values and norms – faculty see how the innovation is appropriate, and fair to faculty implementing it; and (iii) cognitive/cultural institutionalization – characterized by the widespread and shared understanding and acceptance of the innovation through a common framework of meanings (Colbeck, 2002; Scott, 2014). Adopters have a clear sense of ownership pertaining to the innovation, have a nuanced and sophisticated understanding of how to utilize the innovation in a myriad of ways and can interpret their activity as part of normal operations. In addition, the institution itself, develops a growing sense of history and experience with the innovation. Each of these dimensions support one another and may co-occur.

### 3.4 Barriers and facilitators associated with the adoption of either E-Learning or community-focused experiential learning

A review of the literature was conducted to identify the possible facilitators or barriers that are associated with the adoption of either e-learning or community-focused experiential learning^1^ (Table 1). This review served as a framework for investigating what has worked at our own institution in the context of faculty adoption of either of these teaching approaches. It also served as the basis for questionnaire items that were part of a faculty survey, designed to investigate the facilitators and barriers to faculty adoption^2^. Details of the variables that were examined can be found in the methods section of this report. Items from the survey appear in Table 2.

**Table 1:** Variables associated with faculty adoption of e-learning or experiential learning as indicated in the literature.

	e-learning	Experiential Learning (CSL, CBR), Community Engagement Activity and/or Scholarship
Demographics		
Tenure Status/academic rank (non-tenured = ref)		Antonio et al. (2000); Demb & Wade (2012); Glass, Doberneck, & Schweitzer, (2011); Hou & Wilder, (2015)
gender (male = ref)		Bennett et al., (2016); Demb & Wade, (2012); Hou & Wilder (2015); Lunsford and Omae (2011)
Extrinsic Rewards		
Financial incentives (grants)/Extrinsic Rewards	Cook, Ley, Crawford, & Warner (2009); Newton (2003)	Bennett et al., (2016); Glass, Doberneck, & Schweitzer, (2011); Holland (1999); Ward (1998)
Institutional Culture		
Institutional Leadership	Garrison & Vaughan (2012)	Bennett et al. (2016); Demb & Wade, (2012); Holland (1999);
Committee Involvement	Newton (2003)	Ward (1998)
Professional Development opportunities/Teaching support)	Buchanan, Sainter, & Saunders (2013); Cook et al., (2009)	Glass, Doberneck, & Schweitzer (2011) Holland (1999); Hou & Wilder (2015)
Logistical Support/Central organizing structure	Buchanan, Sainter, & Saunders (2013); (Newton, 2003); Zhen et al. (2008); MacKeogh & Fox(2009)	Demb & Wade (2012), Hou & Wilder (2015); Holland (1999)
Faculty Perceptions & Beliefs		
Perceived suitability/risky for student learning Self-efficacy	Buchanan et al. (2013) Horvitz et al., (2015); Zhen et al., (2008)	Hou & Wilder (2015)^15^
Professional Dimensions		
Historical precedence in the discipline		Demb & Wade (2012); Lunsford & Omae, (2011); Holland (1999)
Academic Freedom		
Time commitment for faculty members/ Workload demands	Buchanan, Sainter, & Saunders (2013); Zhen, Garthwait, & Pratt (2008); Cook, Ley, Crawford, & Warner (2009); Newton (2003); MacKeogh & Fox (2009); Visser, (2000)	Demb & Wade (2012); Hou & Wilder, (2015); Lenton et.al. (2016)

*Note.* This table only identifies positive or negative associations (i.e., facilitators or barriers), it does not identify null findings, even though the variable may have been investigated. CSL = Community Service Learning; CBR = Community Based Research

**Table 2:** Items from the survey.

		Reliability	
		e-learning	experiential Learning
Demographics			
Tenured/tenure track vs all others (ref= all others)	What is your current academic rank? (1-contract, 2-assist lecturer, 3-assoc lecturer, 4 - lecturer, 5- senior lecturer, 6-assistant professor, 7-assoc professor, 8 - professor)		
Gender (ref = males)	What is your gender?		
	Female, Male, Non-Binary, Prefer not to say, Prefer to self-describe _________		
Institutional Culture			
Awareness of Institutional Plans (-2 to 2)	*Select the choice that best reflects your opinion. (-2 strongly disagree, 0 Neutral, 2 strongly agree)*		
	I know the contents of the university academic plan, as it pertains to [e-learning / experiential learning]		
	Given the university academic plan, the institution is moving in the right direction to expand the number of [e-learning / experiential learning] courses	.50^1^	.46^1^
Degree of Committee involvement (0-1)			
	*(Select all that apply). In the past 5 years, I have served on committees that advance [e-learning/ experiential learning] at the:*		
	School / department / unit level		
	Faculty level	.74^2^	.63^2^
	University level		
	I have not been involved in committees that advance [e-learning / experiential learning]		
Degree of Professional Development participation on campus, off campus, other (0-1)			
	*(Select all that apply). In the past five years, I have engaged with professional development opportunities pertaining to [e-learning/ experiential learning] through:*		
	On-campus opportunities at York University (e.g., Teaching Commons)		
	Off-campus opportunities	.26^2^	.28^2^
	None of the above		
	Other -- (describe in text box)		
Degree of Logistical Support accessed (0-1)			
	*(Select all that apply). In the past five years, I have worked with:*		
	Learning Technology Services (LTS) /York University Experience Hub		
	Faculty based eLearning support personnel / Faculty based experiential learning coordinator	.46^2^	.53^2^
	I have not engaged with any of the above Other -- (describe in text box)		
Faculty Perceptions/Beliefs			
	*In the items that follow, select the choice that best reflects your opinion regarding [e-learning/ experiential learning] (-2 strongly disagree, 0 Neutral, 2 strongly agree)*		
	[e-learning/ experiential learning] is not appropriate for the courses I teach		
Not effective/risky for student learning (-2 to 2)	Students do not react well to [e-learning / experiential learning].		
	I feel that using new methods such as [e-learning / experiential learning] is risky for student learning	.82^1^	.83^1^
*Self-Efficacy & Confidence*	*In the items that follow, select the choice that best reflects your opinion regarding eLearning. (-2 strongly disagree, 0 Neutral, 2 strongly agree)*		
Aware of teaching methods tools, confident (-2 to 2)	I am aware [of tools available for me to use, to teach online] / [available experiential learning teaching methods]		
	I am aware of specific methods [for teaching fully online/blended courses / [required to work sustainably, with community partners, in developing student projects and/or learning experiences as part of experiential learning courses]	.89^1^	.79^1^
	I am confident in teaching [a fully online/blended learning eLearning course] / [an experiential learning course that involves community partners, to develop and deliver student projects and/or learning experiences]		
Professional Dimensions			
Workload: requires high time commitment/workload (-2 to 2)	*In the items that follow, select the choice that best reflects your opinion. (-2 strongly disagree, 0 Neutral, 2 strongly agree)*		
	I have limited time available to create an [e-learning/ experiential learning] course	.64^1^	.67^1^
	[eLearning/ experiential learning] causes additional workload for faculty members		
Promotion infringes on my academic freedom/bargaining rights (-2 to 2)	The promotion of [e-learning/ experiential learning] violates my academic freedom and/or collective bargaining rights		
No tradition of in my field (-2 to 2)	There is no tradition of [e-learning/ experiential learning] in my field		

*Note.* There were two versions of the survey. One version contained references to e-learning, and the other, to community-focused experiential learning. These terms were defined on each page of the survey.

1 Cronbach’s Alpha

2 KR-20 reliability (internal consistency)

To summarize Table 1, the gender effect for community-focused experiential learning, may be attributed to the fact that this approach has a long history in fields that have been dominated by women (e.g., nursing, education, social work etc.; See Demb and Wade 2012; Lundsford and Omae, 2011). As such, it was important to consider questions pertaining to the historical precedence of the practice within the academic field. Extrinsic rewards (i.e., funding) have been positively associated with the adoption of e-learning as well as experiential learning. Many of the policy and procedure variables associated with institutional culture are in accordance with the recommended actions that have been mentioned in models of organizational change (e.g. committee involvement, professional development, logistical support). Thus, it is not surprising that these variables are associated with the adoption of both e-learning and experiential learning.

## 4 Method

Based on the items in Table 1, two forms of a questionnaire were constructed so that they were nearly identical except that one form focused on questions pertaining to e-learning and the other on community-focused experiential learning. Faculty were asked to review the definitions of e-learning and community focused experiential learning and were then asked to complete the following statement, “In the past five years, I have taught courses that incorporate: (select one) e-learning, community-focused experiential learning, neither or both”. Individuals who selected e-learning, were considered to be “adopters”^3^ and received the e-learning version of the survey. Analogously, those who selected community-focused experiential learning, received the community-focused version of the survey and were considered to be adopters. Of the individuals who answered “neither”, half received the e-learning questionnaire and half received the experiential learning questionnaire and were considered to be “non-adopters”. Of the individuals who taught “both” types, they were asked to choose either e-learning or community-focused experiential learning, based on what was most salient to them. Depending on the version of the survey, definitions of either e-learning or community focused experiential learning appeared on the top of every page. The items from the survey appear in Table 2.

## 5 Questionnaire development

Prior to data collection there were three steps that led up to the questionnaire items that were ultimately used in the analysis for this study. They are listed as follows:

### 5.1 Determination of relevant constructs

As seen in Table 1, a review of the literature enabled the identification of facilitators or barriers for the adoption of either e-learning or community-focused experiential learning.

### 5.2 Development of the questionnaire items

Guiding principles for the creation of the questionnaire items were as follows:

• Questionnaire items mapped onto the constructs in terms of face validity.
• Two main types of questions were asked. One type focused upon agreement or disagreement on various statements, using a five-point Likert scale ranging from -2 (indicating strongly disagree) to 0 (indicating neutral) to +2 (indicating strongly agree). The second type presented a question stem, and then asked to the user to select “all that applied” with a binary response amongst a list of choices. In some cases, it was a simple yes/no answer to a question.
• Space for comments were included (though we did not report these in the present study).

### 5.3 Review of questionnaire items by an expert panel

The initial set of items were created by the first author of this paper and were reviewed by the second author for overall appropriateness and comprehension. Following this, an expert panel reviewed the items. The panel consisted of the director of the teaching and learning center of the university, who is a faculty member and has extensive experience in both e-learning and community-focused experiential learning. The second panel member was a faculty member who has extensive experience in the implementation of e-learning (both fully online and blended). Both judges served on university-level decision making committees pertaining to e-learning or experiential learning or both. The review led to some minor revisions to questionnaire wording and new questionnaire items that were not included in the original questionnaire, reflecting the local context of the institution (e.g., academic freedom).

### 5.4 Ethical review

The questionnaire along with the study proposal (which included methods of online data collection, research design, online informed consent, privacy, use of data and data security, and other required details) was reviewed and approved by the Human Participants Review Sub-Committee, York University's Ethics Review Board (certificate# e2017 – 169). This board conforms to the standards of the Canadian Tri-Council Research Ethics^4^.

### 5.5 Steps following data collection

Two additional steps were undertaken. First, based on the required participants to variables ratio, 12 parameters in the logistic regression analysis could be included (See Participants to Variables Ratio in the Procedure section). This step was consequential, because it meant that we needed to meaningfully capture the information yielded by the questionnaire using only 12 variables that covered the relevant constructs we were interested in. As such, the second step required the creation of summary variables for the purposes of the logistic regression analysis, enabling us to keep the number of variables at 12. To achieve this, scores from items that appeared to be tapping into a common construct as verified by a principal component analysis (PCA), were averaged across items. For example, in the case of questions examining self-efficacy that used a five-point Likert scale, we averaged the scores which ranged from - 2 (strongly disagree) to 0 (neutral) to +2 (strongly agree) to obtain a summary score across the items that tapped into the self-efficacy construct. For items that required a binary response from a series of possible choices (i.e., “select all that apply”), such as degree of committee involvement, we averaged the scores across item choices to obtain a summary score ranging from (0–1) and used this to compare adopters versus non-adopters. In the case of regression, for binary items, we recoded the summary scores (e.g., committee involvement) so that they would receive a score of “1” if the proportion was <0, (to indicate that respondents participated in a committee at least once). To ensure that the resulting sub-scales were reliable, we calculated their reliability either by Cronbach's alpha for sub-scales scored on a continuous scale (i.e., the five -point Likert scale), or by the KR-20 reliability for sub-scales derived from binary scores (i.e., yes, no from a series of choices). For sub-scales that were composed of more than three items, we also checked their dimensionality, using principal component analysis (PCA), to ensure that they were uni-dimensional (Schmitt, 1996; Streiner, 2003; Tavakol and Dennick, 2011).

Below is a description of the variables that were utilized in the analyses that follow. As indicated, some variables were composed of more than one item. In such cases the reliability is reported, along with a description of the original items that were utilized to create the variable. These items can be found in Table 2, along with their reliabilities^5^, and range of scores.

## 6 Demographic characteristics

### 6.1 Tenure status and academic rank

Faculty indicated whether they were contract faculty, assistant lecturer, associate lecturer, senior lecturer, lecturer, assistant professor, associate professor, or professor. In addition, they were also asked to indicate whether they were contract faculty, tenured/continuing, probationary (on the tenure track), or contractually limited^6^. For the analyses, we contrasted tenured/tenure track versus all other categories.

### 6.2 Gender

Faculty selected the gender they identified with (i.e., male, female, non-binary, prefer to self-describe, prefer not to say). For the purposes of the logistic regression analysis we only analysed data from those who selected the male or female category.^7^

## 7 Institutional culture

### 7.1 Institutional leadership

Two items were used for this measure. Faculty indicated the extent to which they knew about the content of the university institutional plans (as highlighted by university leadership). In a separate item, faculty indicated the extent to which they agreed or disagreed to the direction the university was moving in terms of expanding the number of e-learning or experiential learning courses.

### 7.2 Involvement in committees that advance E-learning/experiential learning

Using a binary response, faculty indicated which committees they served on department, Faculty or university in the past five years.

### 7.3 Professional development opportunities

Using a binary response, faculty indicated where they accessed professional development: on-campus, off-campus, or other in the past five years.^8^

### 7.4 Logistical support

Using a binary response, faculty indicated where they sought logistic support^9^ in the past five years.

### 7.5 Financial incentives (grants) & extrinsic rewards

Using a binary response faculty indicated whether they received funding. We did not evaluate whether the amount of funding made a difference, as there were not enough cases across the range of dollar values that were granted for the logistic regression analysis.

## 8 Faculty perceptions/beliefs

### 8.1 Perceived effectiveness for student learning

This measure examines the extent to which the individual perceives that the educational initiative will be effective for student learning. To obtain a summary score, the Likert scores were averaged across three items in Table 2.

### 8.2 Self-efficacy

This measure examines the extent to which the individual perceives that they are able to carry out the educational initiative in their teaching. To obtain a summary score, the Likert scores were averaged across four items in Table 2.

## 9 Professional dimensions

### 9.1 Academic discipline/historical precedence

One item was used to assess this construct using a five-point Likert scale to assess the respondents’ disagreement or agreement with the sentiment expressed in the item (Table 2 “No tradition in my field”).

### 9.2 Academic freedom

One item was included to assess the extent to which a faculty member perceived that the promotion of the teaching initiative (either e-learning or community-focused experiential learning) infringed on their academic freedom or collective bargaining rights, using a five-point Likert scale.

### 9.3 Time commitment for faculty/workload demands

Faculty indicated whether they had enough time to create and e-learning or experiential learning course and whether they perceived it caused additional workload. To obtain a summary score, the Likert scores were averaged across the two items that appear in Table 2 (See workload).

## 10 Procedure

Two weeks prior to the data collection (i.e., November 2017) an email from the Vice-Provost was sent out to all faculty members who taught in the previous academic year between September and June. The email informed faculty about an upcoming survey regarding their engagement with e-learning and experiential learning. The message conveyed that it was important for faculty to complete the survey even if they did not utilize e-learning or experiential learning as a teaching strategy, for the purpose of gaining an understanding of facilitators and barriers to engaging in these different strategies. Additionally, they were informed that while the participation in the survey was completely voluntary, it would be beneficial to fill it out because its results would assist policy and decision makers in terms of identifying barriers to faculty adoption of e-learning/experiential learning and potentially determine ways of mitigating or eliminating such barriers.

Two weeks after initial contact, faculty members were sent an email from the Associate Vice-President Teaching and Learning, containing a link to the survey. The purpose of the survey was restated, and it was emphasized that participation is voluntary. They were also informed that it was important for faculty members to respond, even if they did not utilize e-learning or experiential learning as a teaching strategy. Faculty were asked to complete the survey within three weeks. In addition, faculty were informed that they would be receiving three additional reminder emails if they did not complete the survey. The reminder emails (from the Associate Vice-President's Teaching & Learning staff) were sent once, at the end of each week, and one 48 h prior to the end of the survey, to faculty members who did not respond to earlier requests. For all email communications, faculty members had the option of removing themselves from the email distribution list, if they did not wish to receive additional emails.

At the end of the third week of data collection, the survey was extended by one week, to increase the survey response rate.

## 11 Participants

The participants were contract, contractually limited, tenured or tenure track faculty members at a single large university.

### 11.1 Response rate and analytical sample

A total of 2455 faculty members were contacted via email. Four hundred and twenty-eight faculty members responded to the survey, leading to a response rate of 17.4%. Two respondents failed to provide answers to any of the questions, resulting in an analytical sample of 426. The pattern of respondents was compared to the official count of the number of faculty employed in the university as a function of Faculty affiliation. A chi-square goodness of fit test revealed that the pattern of respondents was proportional to the official counts as a function of Faculty affiliation χ^2^ (9, *N* = 419) = 12.52, *p* = .186. Furthermore, a chi-square goodness of fit test revealed that the pattern of respondents was also proportional to the official counts of faculty as a function of their gender χ^2^ (1, *N* = 426) = .001, *p* = .973. However, based on the proportion of contract faculty (including contractually limited appointments) versus tenured and tenured faculty, a higher number of tenured and tenure track faculty responded to the survey compared to contract faculty, as indicated by the chi-square goodness of fit test χ^2^ (1, *N* = 404) = 71.58, *p* = .000.

It is important to keep in mind the number of adopters was likely oversampled in this study. This is despite our encouragement of non-adopters to participate in the survey. Unfortunately, there was no way of independently estimating the expected proportion of faculty members who adopted e-learning or experiential learning compared to those who did not. That being said, the study did indeed have equal numbers of adopters versus non adopters as indicated by a non-significant chi-square goodness of fit test assuming equiprobabilty across both groups on all measures. (See the results section under “demographic characteristics”, reported separately for e-learning and community-focused experiential learning).

### 11.2 Participants to variables ratio: a note on the number of parameters in the regression model in reference to sample size

In this study we examined responses from 85 non-adopters and 119 adopters of e-learning, and 92 non-adopters and 95 adopters of community-focused experiential learning. For the purposes of the logistic regression analysis, Peduzzi et al., (1996; cited in Hosmer et al., 2013), suggests a ratio of 10 events (or non-events) per parameter for a binary logistic regression. However, Vittinghof & McCulloch, (2006; cited in Hosmer et al., 2013), suggest that this criteria may be too conservative, and indicate that a range between 5 - 9 events per parameter is acceptable. As such, the present study utilizes a logistic regression model of 12 parameters based on a ratio of at least 7 events per parameter.

## 12 Results

### 12.1 E-learning: comparison of adopters versus non-adopters

#### 12.1.1 Demographic characteristics

Table 3 lists the demographic characteristics of the respondents that were examined in the study: Appointment type (tenured/tenure track versus all others) and gender (females versus males). Based on the chi-square goodness of fit test, assuming equiprobability (i.e., equal proportions) across adopters versus non-adopters for the overall expected frequencies, there were no significant differences in terms of the number of e-learning adopters and non adopters for appointment type χ^2^ (1, *N* = 216) = 1.852, *p* = .174; or gender χ^2^ (1, *N* = 203) = 2.172, *p* = .141. That is, within the analytical sample there was roughly the same number of adopters versus non-adopters for e-learning, regardless of appointment type and gender.

**Table 3:** Frequency of faculty members who taught at least one e-learning (blended or fully online) course (adopters) versus those who did not (non-adopters) as a function of demographic characteristics, and funding.

	e-learning non-adopters	e-learning adopters
	*n*	*n*
Appointment type		
tenure track/tenured	67	73
all others	31	45
totals	98	118
Gender		
F	47	63
M	44	49
totals	91	112
Funding		
no	99	97
yes	1	21
totals	100	118

To see if the frequencies varied between adopters and non-adopters as a function of the levels of the demographic variables (i.e., appointment type: tenured/tenure track vs all others, gender: male vs female), a chi-square test of independence was conducted. Across both variables, a non-significant difference was obtained, indicating that there was independence between adopters versus non-adopters by appointment type χ^2^ (1, *N* = 216) = .993, *p* = .319; or gender χ^2^ (1, *N* = 203) = .428, *p* = .513. In other words, based on a headcount, there is no relation between e-learning adoption as a function of the levels of the demographic variables examined (i.e., no difference in counts for tenured/tenure track vs all other faculty; and no difference in counts for male vs female).

#### 12.1.2 Extrinsic rewards/funding

There appears to be an association between receiving funding, and the faculty member adopting e-learning (Table 3). For faculty members who did not receive funding, there appears to be a very small difference in the frequency of e-learning adoption versus non-adoption. In contrast, for faculty members who received funding, there is a dramatic difference in the frequency of e-learning adoption versus non-adoption. Indeed, a chi-square test of independence confirms that these two variables are indeed dependent χ^2^ (1, *N* = 218) = 16.831, *p* = .000. In other words, based on a headcount, the adoption of e-learning depends on receiving funding: Differences favouring adopters emerge when funding is present, but such differences are absent, when funding is not present. As such, funding appears to be associated with e-learning adoption. (Though, note the logistic regression results to follow).

#### 12.1.3 Institutional culture variables

Adopters agreed more strongly that they were aware of institutional plans, compared to non-adopters (Table 4). Adopters also indicated that they had more committee involvement pertaining to e-learning and accessed logistical support to a higher degree than non-adopters. Although adopters indicated that they accessed professional development to a numerically higher degree, compared to non-adopters, this was not significantly different.

**Table 4:** Comparison of faculty members have taught at least one e-learning (Blended or Online Course) in the last 5 years (adopters) with those who have not (non-adopters).

	e-learning non-adopters			e-learning adopters			*F*	*sig*	*eta sq*	
	Mean	N	Std. Deviation	Mean	N	Std. Deviation				
Institutional Culture										
Awareness of Institutional Plans (-2 to 2)	-.19	85	.78	.36	118	.91	20.33	.000	.092	∗∗∗
Degree of e-learning Committee involvement (0-1)	.12	85	.25	.24	116	.32	9.05	.003	.044	∗∗
Degree of e-learning PD participation on campus, off campus, other (0-1)	.42	30	.15	.44	93	.16	.30	.582	.003	n.s.
Degree of eLearning Logistical Support accessed (0-1)	.27	84	.07	.37	113	.14	34.46	.000	.150	∗∗∗
Faculty Perceptions/Beliefs										
Not appropriate/risky for student learning (-2 to 2)	.43	85	.92	-.88	118	1.04	85.73	.000	.299	∗∗∗
*Self-Efficacy & Confidence*										
Aware of teaching methods, confident (-2 to 2)	.11	84	.96	.98	118	.99	39.19	.000	.164	∗∗∗
Professional Dimensions										
Workload: e-learning requires high time commitment/workload (-2 to 2)	1.21	84	.81	.79	118	1.03	9.97	.002	.047	∗∗
Promotion of e-learning infringes on my academic freedom/bargaining rights (-2 to 2)	.00	84	1.32	-.79	118	1.24	18.83	.000	.086	∗∗∗
No tradition of e-learning in my field (-2 to 2)	.14	85	1.18	-.41	117	1.28	9.77	.002	.047	∗∗

*Note.* ∗∗∗p < .001, ∗p < .01 , ∗p <.05; (-2 to 2) = five point Likert scale where -2 = strongly disagree, -1 = disagree, 0 = neutral, 1 = agree, 2 strongly agree ; (0 -1) = a binary scale, 1 = yes, 0 = no; PD = professional development.

#### 12.1.4 Faculty perceptions/beliefs

Adopters disagree more strongly that e-learning is not effective, or that e-learning as a new teaching method is risky for student learning compared to non-adopters (Table 4). In contrast, adopters agreed more strongly with statements indicating that they are aware of e-learning teaching methods and are more confident compared to non-adopters.

#### 12.1.5 Professional dimension variables

Non-adopters agreed more strongly that e-learning requires a high time commitment/workload, compared to adopters (Table 4). Non-adopters also agreed more strongly that there was no tradition of e-learning in their field, compared to adopters. On the other hand, adopters disagreed more strongly that the promotion of e-learning infringes upon their academic freedom/bargaining rights, while non-adopters were neutral.

### 12.2 Variables associated with e-learning adoption: logistic regression findings^10^

#### 12.2.1 Demographic characteristics

Faculty members who are tenured or on the tenure track are less likely to engage in teaching a blended or fully online course (Table 5). In fact, faculty who are not tenured are 4.01 times more likely to engage in teaching an e-learning course. Note that although this pattern is not immediately apparent in the context of the raw frequencies (i.e., head-count) presented in Table 3, it becomes apparent if one compares the difference scores (as a proportion); that is the difference between adopters versus non-adopters for non-tenured faculty with the difference for tenured faculty. The difference is very small for tenured faculty (i.e., 6 out of 140, or 4%) compared to non-tenured faculty (i.e., 14 out of 76, or 18%). Hence, knowing that an individual is non-tenured is diagnostic, and suggests that they are more likely to adopt e-learning, whereas knowing somebody is tenured, is less diagnostic. So, from this perspective the regression results are indeed consistent with the analysis comparing adopters versus non-adopters. Based on Table 5, gender is not predictive in whether faculty will utilize e-learning as a teaching strategy. The latter finding is consistent with that reported in Table 3, the comparison of adopters versus non-adopters.

**Table 5:** Final Logistic Regression model predicting faculty members' adoption of e-learning (Blended or Fully online) courses.

		B	S.E.	Wald	Sig.		Exp(B)	95% C.I. for EXP(B)	
							odds ratio	Lower	Upper
Demographics									
	Tenured/tenure track vs all others (ref= all others)	-1.39	.52	7.21	.007	∗∗	.25	.09	.69
	Gender (ref = males)	-.36	.43	.72	.397		.70	.30	1.61
Extrinsic Rewards: Funding									
	I received funding to create an e-learning course (ref = no)	.13	.59	.05	.824		1.14	.36	3.63
Institutional Culture									
	Awareness/Agreement with direction of institutional plans (5-pt Likert scale)	.45	.28	2.55	.111		1.56	.90	2.71
	Committee Involvement, at least once (ref = none)	.57	.51	1.28	.258		1.77	.66	4.79
	Professional Development participation, at least once (ref = none)	1.21	.47	6.79	.009	∗∗	3.36	1.35	8.38
	Accessed Logistic Support, at least once (ref = none)	.66	.97	.46	.497		1.93	.29	12.96
Faculty Perceptions/Beliefs (5-pt Likert scale)									
	Perceive e-learning is not suitable, students do not react well and is risky for learning	-1.00	.26	14.64	.000	∗∗∗	.37	.22	.62
	Self-efficacy: Aware of teaching methods, required steps, confident	.33	.24	1.94	.164		1.40	.87	2.23
Professional Dimensions (5-pt Likert scale)									
	Requires high time commitment/workload	-.47	.25	3.49	.062	+	.63	.38	1.02
	The promotion of eLearning violates my academic freedom and/or collective bargaining rights	.26	.19	1.85	.174		1.29	.89	1.86
	There is no tradition of e-learning, in my field	.10	.18	.32	.572		1.11	.77	1.59
	Constant	-.38	1.10	.12	.731		.68		

Note. ∗∗∗*p* < .001, ∗*p* < .05 , +*p* < .10; pt = point.

#### 12.2.2 Extrinsic rewards/funding

It appears that funding does not predict whether a faculty member will engage in teaching a blended or online course (Table 5). This contrasts with that reported in Table 3. It is important to note that this variable was indeed significant, if all the subsequent variables were not included in the regression model (or is considered as the only predictor). Direct evidence of the importance of funding can be seen in the first four columns of Table 6, the overall summary of the logistic model in its ability to predict faculty adoption. The addition of the funding variable significantly adds to the prediction of e-learning adoption, over and above that predicted by a model that only includes demographics as a predictor. However, in the context of the full regression model, which simultaneously considers the effects of all other variables, funding is non-significant.

**Table 6:** Assessment of Model: How much each cluster of variables add to prediction of e-learning (Blended or Fully online) course adoption, R- Square, Model Fit, and percentage of cases correctly classified.

Model summary	Chi Sq.	df	sig	Pseudo R-Sq Indices			H-L model Fit			% Cases classified
				C&S R-Sq.	Negelkerke R-Sq.	McFadden R-Sq.	Chi Sq	df	p-value	
Demographics	1.59	2	.451	.01	.01	.01	.21	2.00	.90	56.4
Extrinsic Rewards: Funding	19.39	1	.000	.11	.14	.08	.04	4.00	1.00	63.3
Institutional Culture	43.00	4	.000	.29	.39	.25	8.37	8.00	.40	72.3
Faculty Perceptions/Beliefs	34.80	2	.000	.41	.55	.38	6.88	8.00	.55	80.3
Professional Dimensions	5.11	3	.164	.43	.57	.40	5.07	8.00	.75	80.3
Overall	103.89	12	.000							

*Note.* Sq = Square, C&S = Cox & Snell, H-L = Hosmer & Lemeshow

#### 12.2.3 Institutional culture variables

Participation of professional development (either off campus, on campus or other) at least once, was the only predictor of whether a faculty member will engage in e-learning as a teaching strategy (Table 5). Faculty were 3.36 times as likely to engage in e-learning as a teaching strategy if they engaged in e-learning professional development at least once.

#### 12.2.4 Faculty perceptions/beliefs as predictors of e-learning adoption

The perception that e-learning, as a new method is risky and not effective for student learning appeared to be negatively related to faculty members adopting e-learning as a teaching strategy (Table 5). The odds of not engaging was 2.70 times greater for each unit increase in their agreement with this item on the five-point Likert scale. In contrast, self-efficacy (i.e., awareness of teaching methods, confidence) appeared to be positively related to e-learning adoption, though this effect was non-significant. This finding is consistent with what was seen in Table 4 in terms of the mean rating scores of adopters versus non-adopters.

#### 12.2.5 Professional dimension variables

The perception that e-learning requires a high time commitment/workload was negatively related to e-learning adoption (Table 5). Faculty members were 1.60 times as likely of not adopting e-learning, for each unit increase in their agreement with this item on the five-point Likert scale. This effect was marginally significant (*p* = .06). No other professional dimension variables were significant.

### 12.3 Model summary: the relative importance of the categories of variables predicting e-learning adoption

The full model significantly adds to the prediction of e-learning adoption, over and above the null model χ^2^ (12, *N* = 188) = 103.89, and correctly classifies 80.3% of the cases into adopters and non-adopters based on the variables examined (Table 6). The non-significant Hosmer-Lemeshow values suggest that this model adequately represents the pattern of the data (i.e., the predicted values fit or are sufficiently close to the observed values). Values of pseudo R-square are presented in columns four, five and six of Table 6, and enable the assessment of how much variance is explained by each category of variables.^11^ Based on the McFadden pseudo R-square, it appears that institutional culture, is the most important category of variables in predicting faculty adoption of e-learning (predicting 17% of the variance in the prediction of e-learning adoption), followed by faculty perceptions/beliefs (explaining 14%). Extrinsic reward was the third most important category of variables (explaining 7% of the variance). In contrast, demographics and professional dimensions explain a very small amount of variance in the prediction of the e-learning adoption (i.e., 1 and 2 percent respectively). Thus, if our goal were to develop the most concise model to predict e-learning adoption, one would focus on variables captured by institutional culture, faculty perceptions/beliefs, and funding.

### 12.4 Community-focused experiential learning: adopters versus non-adopters

#### 12.4.1 Demographic variables

A chi-square goodness of fit test (Table 7), which assumes equiprobability for the overall expected frequencies for adopters versus non-adopters, revealed no differences in terms of appointment type χ^2^ (1, *N* = 188) = .085, *p* = .77; and gender χ^2^ (1, *N* = 186) = .022, *p* = .883.

**Table 7:** Frequency of faculty members who taught at least one community-focused experiential learning course (adopters) versus those who did not (non-adopters), as a function of demographic characteristics, and funding

	Ex Ln non-adopters	Ex Ln adopters
	*n*	*n*
Appointment type		
tenure track/tenured	66	74
all others	26	22
totals	92	96
Gender		
F	37	59
M	51	33
totals	92	94
Funding		
no	108	85
yes	2	12
totals	110	97

Note. Ex Ln = experiential learning

To examine whether the frequencies varied between adopters and non-adopters as a function of the levels of the demographic variables, a chi-square test of independence was conducted. According to the analysis there was no relation between adopters and non-adopters in terms of appointment type χ^2^ (1, *N* = 188) = .706, *p* = .401. In other words, there was roughly the same number of adopters as non-adopters within the category of tenured faculty. Similarly, there was roughly the same number of adopters versus non-adopters within the category of “all other faculty” (i.e., including contract faculty). However, there was evidence of an association between adopters versus non-adopters and gender χ^2^ (1, *N* = 186) = 8.814, *p* = .003. Indeed, a pattern can be seen, where amongst female faculty, there are more experiential learning adopters compared to non-adopters. In contrast, there is a reverse pattern for male faculty, where there are more experiential learning non-adopters compared to adopters.

#### 12.4.2 Extrinsic rewards/funding

There appears to be evidence of an association between receiving funding, and the faculty member adopting community-focused experiential learning (Table 7). For faculty members who did not receive funding, there appears to be a greater number of individuals who did not adopt community-focused experiential learning, compared to those who did. In contrast, for faculty members who received funding, the opposite pattern was apparent: more individuals adopted community-focused experiential learning courses compared to those who did not. Indeed, a chi-square test of independence confirms that these two variables are indeed dependent χ^2^ (1, *N* = 207) = 9.103, *p* = .003. (Though, note the results from the logistic regression below).

#### 12.4.3 Institutional culture variables

Adopters gave significantly stronger ratings in terms of their awareness of institutional plans. Adopters had a higher degree of committee involvement pertaining to experiential learning and accessed logistical support to a higher degree than non-adopters (Table 8). Adopters also accessed professional development to a higher degree, than non-adopters, though this effect was marginally significant.

**Table 8:** Comparison of faculty members have taught at least one Community-focused Experiential learning course in the last 5 years (adopters) with those who have not (non-adopters).

	Ex Ln non- adopters			Ex Ln adopters			*F*	*sig*	*eta sq*	
	*Mean*	*N*	*Std. Deviation*	*Mean*	*N*	*Std. Deviation*				
Institutional Culture										
Awareness of Institutional Plans (-2 to 2)	.19	93	.79	.85	96	.80	32.14	.000	.147	∗∗∗
Degree of Ex Ln Committee involvement (0-1)	.12	92	.25	.26	96	.30	12.23	.001	.062	∗∗∗
Degree of Ex Ln PD participation (0-1)	.38	24	.11	.44	70	.16	3.33	.071	.035	+
Degree of Ex Ln Logistical Support accessed (0-1)	.17	89	.04	.22	94	.10	19.25	.000	.096	∗∗∗
Faculty Perceptions/Beliefs										
Not appropriate/risky for student learning (-2 to 2)	-.39	93	.87	-1.50	96	.76	88.29	.000	.321	∗∗∗
Self-Efficacy & Confidence										
Aware of teaching methods tools, confident (-2 to 2)	-.27	93	1.00	.95	96	.86	79.96	.000	.300	∗∗∗
Professional Dimensions										
Workload: Ex Ln requires high time commitment/workload (-2 to 2)	1.12	93	.82	.52	96	1.12	17.83	.000	.087	∗∗∗
Promotion of Ex Ln infringes on my academic freedom/bargaining rights (-2 to 2)	-.49	91	1.09	-1.32	96	1.03	28.55	.000	.134	∗∗∗
No tradition of Ex Ln in my field (-2 to 2)	-.32	93	1.32	-1.42	96	.91	44.12	.000	.191	∗∗∗

Note. ∗∗∗p < .001, ∗p < .01 , ∗p <.05, +p <.10; (-2 to 2) = five point rating scale where -2 = strongly disagree, -1 = disagree, 0 = neutral, 1 = agree, 2 strongly agree; 0 -1) = a binary scale, 1 = yes, 0 = no; Ex Ln = experiential learning; PD = professionl development.

#### 12.4.4 Faculty perceptions/beliefs

Adopters agree to a greater extent that there is a lack of funding/support for experiential learning compared to non-adopters (Table 8). Adopters disagree to a greater extent that experiential learning is not effective or risky (as a new teaching method) for student learning compared to non-adopters, who disagree to a lesser extent. Adopters more strongly endorse statements indicating that they are aware of experiential learning teaching methods, and are more confident, compared to non-adopters. No other significant differences between adopters and non-adopters were apparent in this set of measures.

#### 12.4.5 Professional dimension variables

Non-adopters agreed more strongly that experiential learning requires a high time commitment/workload, compared to adopters (Table 8). Adopters disagreed more strongly that there was no tradition of experiential learning in their field, compared to adopters. Adopters disagreed that the promotion of experiential learning infringes upon their academic freedom/bargaining rights, while non-adopters were neutral.

### 12.5 Variables associated with community-focused experiential learning adoption: logistic regression findings

#### 12.5.1 Demographic variables

The only variable that is predictive that a faculty member has taught a community-focused experiential learning course is gender (Table 9). Based on the analysis, it appears that female faculty members are 2.98 times as likely as male faculty members to engage in the teaching of at least one community-focused experiential learning course. This finding is consistent with that found in the comparison of adopters versus non-adopters.

**Table 9:** Final Logistic Regression model predicting faculty members' adoption the teaching of community-focused experiential learning course.

		B	S.E.	Wald	Sig.		Exp(B)	95% C.I. for EXP(B)	
							Odds ratio	Lower	Upper
Demographics									
	Tenured/tenure track vs all others (ref= all others)	.16	.54	.08	.773		1.17	.41	3.35
	Gender (ref = males)	1.09	.48	5.27	.022	∗	2.98	1.17	7.58
Extrinsic Rewards									
	Received funding to create a community-focused Ex Ln course (ref = no)	.84	1.11	.57	.450		2.32	.26	20.45
Institutional Culture									
	Awareness/Agreement with direction of institutional plans (5-pt Likert scale)	-.32	.35	.83	.361		.73	.37	1.44
	Committee Involvement, at least one (ref = none)	1.16	.52	5.04	.025	∗	3.19	1.16	8.77
	Professional Development participation, at least once (ref = none)	.78	.52	2.22	.136		2.18	.78	6.05
	Accessed Logistic Support, at least once (ref = none)	1.60	.82	3.79	.051	+	4.94	.99	24.62
Faculty Perceptions/Beliefs (5-pt Likert scale)									
	Perceive Ex Ln is not suitable, students do not react well and is risky for learning	-1.02	.31	10.50	.001	∗∗∗	.36	.20	.67
	Self-efficacy: Aware of teaching methods, required steps, confident	.89	.25	12.28	.000	∗∗∗	2.43	1.48	3.98
Professional Dimensions (5-pt Likert scale)									
	Requires high time commitment/workload	-.05	.25	.03	.853		.96	.59	1.55
	The promotion of Ex Ln violates my academic freedom and/or collective bargaining rights	-.31	.24	1.64	.201		.73	.46	1.18
	There is no tradition of EE, in my field	-.55	.24	5.17	.023	∗	.58	.36	.93
	Constant	-4.00	.80	24.94	.000	∗∗∗	.02		

Note. ∗∗∗*p* < .001, ∗*p* < .05 , +*p* < .10; pt = point; Ex Ln = experiential learning.

#### 12.5.2 Extrinsic rewards/funding

It appears that funding does not predict whether a faculty member will engage in teaching a community-focused experiential learning course (Table 9). This finding is in contrast with that discussed above, where community-focused experiential learning adoption varies as a function of the presence of funding (Table 7). As in the case for e-learning, it is interesting to note that this predictor is only significant when all the subsequent variables were not included in the model (or is considered as the only predictor). That is, it is only significant if other variables are not held constant or controlled for. Furthermore, direct evidence of the importance of funding can be seen in the first four columns of Table 10. The addition of the funding variable significantly adds to the prediction of community-focused experiential learning adoption, over and above that predicted by a model that only includes demographics as a predictor. However, given that the current model simultaneously considers the effect of all the other variables, funding is non-significant in this context.

**Table 10:** Assessment of Model: How much each cluster of variables add to the prediction of community-focused experiential learning course adoption, R- Square, Model Fit, and percentage of cases correctly classified.

Model summary	Chi Sq	df	sig	C&S R- Sq.	Pseudo R-Sq Indices		H-L model Fit		p-value	% Cases classified
					Negelkerke R-Sq.	McFadden R- Sq.	Chi Sq	df		
Demographics	10.31	2	.006	.05	.07	.04	.61	2.00	.74	61.3
Extrinsic Rewards: Funding	6.43	1	.011	.09	.12	.07	.36	3.00	.95	61.3
Institutional Culture	48.66	4	.000	.30	.40	.25	3.85	8.00	.87	75.8
Faculty Perceptions/Beliefs	54.77	2	.000	.48	.64	.45	6.68	8.00	.57	80.6
Professional Dimensions	7.81	3	.050	.50	.66	.50	4.92	8.00	.77	82.8
Overall	127.97	12	.000							

Note. Sq = Square, C&S = Cox & Snell, H-L = Hosmer & Lemeshow

#### 12.5.3 Institutional culture variables

Committee involvement appears to be a significant predictor of experiential learning adoption (Table 9). According to the regression model, faculty members who are involved in at least one committee are 3.19 times as likely to teach a community-focused experiential learning course as those who are not involved. Faculty members who access logistic support are 4.94 times as likely to engage in teaching using community-focused experiential learning compared to those who do not. This latter result was marginally significant (*p* = .051).

#### 12.5.4 Faculty perceptions/beliefs

The perception that community-focused experiential learning as a new teaching method is risky, and not effective for student learning is negatively related to the adoption of community-focused experiential learning as a teaching strategy (Table 9). The odds of not engaging with experiential learning was 2.76 times greater for each unit increase in their agreement with the perception that experiential learning was risky/not effective for student learning, on a five-point Likert scale. In contrast, a faculty members’ self-efficacy is positively related to their adoption of community-focused experiential learning. The odds of faculty adoption of community-focused experiential learning increases by 2.43 times, for each unit increase in agreement with the statements measuring self-efficacy, on a five-point Likert scale.

#### 12.5.5 Professional dimension variables

The perception that there is no tradition of experiential learning in the faculty members field, is negatively related to the faculty members adoption of community-focused experiential learning (Table 9). The odds of not adopting increases by 1.74 times for each unit increase in agreement with the notion that there is no tradition of experiential learning, based on a five-point Likert scale.

### 12.6 Model summary: the relative importance of the category of variables predicting community-focused experiential learning adoption by faculty

The full model significantly adds to the prediction of e-learning adoption, over and above the null model χ^2^ (12, *N* = 186) = 127.97, and correctly classifies 82.8% of the cases into adopters and non-adopters based on the variables examined (Table 10). The non-significant Hosmer-Lemeshow values suggest that this model adequately represents the pattern of the data (i.e., the observed values fit the predicted values). Values of pseudo R-square are presented in columns four and five of Table 10. Based on the McFadden change of R-square from one category of variables to the next suggests that both institutional culture, and faculty perceptions/beliefs are the most important category of variables in predicting faculty adoption of community-focused experiential learning (each explaining 19% of the variance). Other variables explained the remaining variance: Professional dimensions explained 5% of the variance, demographics explained 4% and extrinsic rewards explained 3%. It is interesting to note that across e-learning and experiential learning the top two sets of variables in terms of explaining variance, were institutional culture and faculty perceptions/beliefs.

## 13 Discussion

Not all actions are equal in terms of increasing the odds of innovation adoption. Across both teaching approaches, we demonstrated the primary importance of an institutional culture that promotes teaching innovation through an institutional plan, and committee involvement and builds capacity for its adoption through professional development and logistical support. In reference to models of organizational change, the results highlight the importance of the pre-implementation phase (e.g., Armenakis and Harris, 2002; Armenakis et al., 1993; Haque et al., 2016) which in part, seeks to mitigate resistance amongst members through a persuasion strategy and individual involvement. Indeed, adopters were more likely to be aware of institutional plans and were more likely to be involved in committees compared to non-adopters. Furthermore, the regression analysis, demonstrated that committee involvement was positively associated with the probability of the adoption of community-focused experiential learning. Recall that Armenakis and his colleagues (Armenakis and Harris, 2002; Armenakis et al., 1993) indicated that the change message by leadership should address amongst other things, self-efficacy (i.e., that faculty will be able to adopt the teaching innovation, and will have a way to do so). Accordingly, it was evident that a greater proportion of experiential learning adopters engaged in professional development compared to non-adopters, and a greater proportion of experiential learning or e-learning adopters accessed logistical support compared to non-adopters. Furthermore, the regression analyses revealed that professional development was positively associated with the adoption of e-learning, and that committee involvement and accessing logistic support, was positively associated with the adoption of community-focused experiential learning. Thus, from the perspective of the institution, some combination of the elements listed under the rubric of institutional culture (Table 1) are necessary to set the stage for change and serve to increase the likelihood of individual faculty adoption of either approach. Clusters of variables within this combination align with the pre-implementation phase (Armenakis et al., 1993; Armenakis and Harris, 2002), in terms of the institutional promotion of the innovation (i.e., raising faculty awareness and increasing the individual level of agreement within each faculty member regarding institutional plans, fostering individual faculty involvement so that they are part of making decisions about the change), as well as the faculty provision of support (thereby raising self-efficacy and building institutional capacity). Indeed, the regression analyses revealed that the institutional culture variables explained the greatest amount of variance in the adoption of either e-learning (Table 6) or community-focused experiential learning (Table 10).

A second and equally important factor is how faculty view institutional actions as manifested in their perceptions and beliefs. The analyses revealed that faculty are more likely to adopt a given teaching innovation, if they have a strong positive perception regarding the direction of institutional plans, the effectiveness of the proposed teaching approach, and a positive sense of self-efficacy (i.e., they know what to do), and view the teaching innovation as being appropriate and not risky as a new method for student learning. These findings are consistent with literature that indicates that resistance can be mitigated if individuals hold positive views about a proposed change (Elias, 2009), so long as the change will positively impact themselves and the organization as a whole (Armenakis et al., 2007). In addition, theories of technology adoption highlight the importance of self-efficacy in terms of its implementation (Horvitz et al., 2015; Zhen et al., 2008), and its perceived effectiveness for student learning for its adoption as a teaching practice (Buchanan et al., 2013). The present study extends this notion to the adoption of community-focused experiential learning. Indeed, faculty perceptions and beliefs explained almost an equal amount of unique variance in the adoption of e-learning or community-focused experiential learning as a teaching practice, independent of the institutional culture variables. This pattern of findings highlights the importance of the faculty perspective, which – in this case, complements the institutional actions of promoting the teaching innovation and supporting faculty through capacity building. The regression analyses underscore that faculty themselves, have agency in deciding whether to adopt, as evidenced by their perceptions and beliefs, independent of the actions that the institution embarked upon to promote innovation adoption. Indeed, researchers posit that the locus of organizational change, occurs at the level of the individual (Haque et al., 2016; Thompson, 2019). That is, if individuals do not change, then organizations will not either (Schneider and Guzzo, 1996). Within this context, the role of leadership is to get individuals psychologically and behaviorally ready to make change (Elias, 2009; Haque et al., 2016; Weiner et al., 2008).

Funding has been advocated by researchers as a mechanism to enhance the adoption of community-focused experiential learning (Bennett et al., 2016; Demb and Wade, 2012; Glass et al., 2011; Holland, 1999; Ward, 1998) and for e-learning (Cook et al., 2009; Newton, 2003). Indeed, funding serves a number of important symbolic functions for the institution and the faculty member (Jenkins et al., 1998). From the perspective of the institution, it can be a way of highlighting, validating, and legitimizing the teaching activity, that the institution wishes to promote and develop. From the perspective of the faculty member, receiving funding can bestow a sense of honour and pride particularly if it is granted on a competitive basis and it is announced in a public fashion (Gallus and Frey, 2016). It also acknowledges the additional work that faculty members must undertake to implement the teaching practice.

On the surface, the present study demonstrates that funding appears to be a diagnostic indicator of whether a faculty member will adopt the teaching innovation. For example, based on a head count, faculty who received funding were (obligated and) likely to adopt the innovation by a large margin. Whereas amongst those who did not receive funding, there was a roughly even chance of adoption (that is, a 50-50 chance of e-learning adoption and a 55-44 chance favoring experiential learning adoption). However, the regression analyses, points to a different conclusion. That is, funding was not significantly associated with adoption behavior, and explained a small amount of variance in adoption relative to the institutional culture variables, and faculty perceptions and beliefs (i.e., 7 % for e-learning and 3 % for community-focused experiential learning). In other words, funding is not a significant motivator for either approach, after considering other factors, such as institutional culture or faculty perceptions and beliefs.

Although it is beyond the scope of the current study, the above pattern of findings is intriguing, given the fact that the behavioral economics literature shows that payment for a desired activity increases only under some circumstances and in others, decreases (Kamenica, 2012). According to this research, finding the optimal amount of payment is a challenge. For example, the marginal returns of payment will decrease the more financially secure an individual is (e.g., tenured faculty). Also, paying too much may cause an individual to be in danger of being overwhelmed due to the imposed obligation, in other words “freeze” or “choke under pressure” (Gallus and Frey, 2016). Payment may crowd out desired behavior that is intrinsically motivated (Jenkins et al., 1998). Payment is effective for tasks that are perceived to be difficult or boring (Gallus and Frey, 2016; Gneezy et al., 2011), and thus offering payment may in turn, alter the perception of the task itself (Kamenica, 2012). There is also the risk that desired behaviors may become transactional, that is, individuals may only do the bare minimum activity in exchange for payment, and behavior may stop, once payment stops (Gneezy et al., 2011).

Given the inherent challenges of utilizing extrinsic sources of motivation, it is useful to consider the work of Stupnisky et al. (2018) who examined faculty adoption of best practices in teaching using a more nuanced range of motivation (i.e., beyond the intrinsic vs extrinsic binary). Based on Ryan and Deci’s (2000; 2017) self-determination theory, Stupnisky et al. (2018) conceptualized motivation into three broad categories based on the extent to which the motivation for engaging in a behavior is internalized – external motivation, introjected motivation and autonomous regulation. Of relevance to the present investigation is the third category, which is based on a desire for engaging in a behavior that is consistent with one's identity, beliefs and values. Stupnisky et al. (2018) demonstrated that autonomous regulation is supported by satisfying three psychological needs as outlined by self-determination theory (Ryan & Deci, 2000, 2017). These are based on an individuals' need for a sense of (i) competence (e.g., by providing faculty ways in which they can develop their skills through professional development or communities of practice, and through recognition of good practice); (ii) relatedness; that is, close and secure bonds with significant others (e.g., by networking with mentors, and a means of ensuring that adopters are not isolated, and verbal support by institutional leadership); and (iii) autonomy; by ensuring that faculty members have options (including non-participation or non-adoption) and a freedom of choice (e.g., by upholding long-standing academic values such as academic freedom), thereby fostering willingness, self-determination, and volition. The underlying assumption is that internally motivated behavior (such as autonomous regulation) is the most enduring and sustainable behavior, whereas externally motivated behavior is less enduring, because it requires the presence of an outside force. In the latter case, behavior will stop, once the external control stops (e.g., funding ends or, social pressure stops).

In relation to the present study, it is interesting to note the very conditions that support autonomous regulation as empirically verified by Stupnisky et al. (2018) and more recently by others (see Averill and Major, 2020), are indeed similar to the set of variables most strongly associated with adoption. These are the institutional culture variables which support faculty in their need for competence (e.g., professional development, & logistical support), and reinforced by the perception and beliefs variables, which capture the need for competence as manifested in faculty members’ sense of self-efficacy. Similarly, the role of relatedness in supporting autonomous regulation, can be captured by institutional culture variables such as committee involvement and professional development workshops (that are face-to-face), because they provide opportunities for faculty to connect with others who are interested in similar topics.

The current study examined additional factors that were hypothesized to be associated with adoption such as demographic characteristics (e.g., tenure status, gender) as well as professional dimensions (e.g., time commitment/workload, academic freedom, historical precedence). Ultimately, these factors explained a small amount of variance (e.g., between 4 and 5% for community-focused experiential learning and between 1 and 2% for e-learning) compared to institutional culture, and faculty perceptions and beliefs (which together explained 26% and 32% of the variance in e-learning and community-focused experiential learning adoption). The finding that gender is associated with the adoption of community-focused experiential learning is consistent with the literature and may represent the fact that many of the subject areas that community-focused experiential learning are those that are dominated by female faculty (e.g., education, nursing, social work)^12^. Furthermore, historical precedence within the discipline was associated with adoption of community-focused experiential learning. Although, e-learning was not associated with gender, more research is required to reconcile why recent reports indicate a gender effect favouring female faculty (e.g., Jackson, 2019).

With respect to e-learning adoption, being tenured was negatively associated, but not for community-focused experiential learning. The reason for this finding is unclear and furthermore it has not been detected in past literature. One possible explanation for this finding may be that tenured faculty view e-learning to require a large time-commitment and contribute to a higher workload as seen in the regression analyses. However, the same concerns for workload appear for faculty who are implementing community-focused experiential learning, and in the latter case we do not see a tenure status effect. A such, we recommend that this result be interpreted with caution, and require that it be replicated. The possibility that this result may be an artefact is explored in the section that follows (See Strengths, Weaknesses and Future Considerations).

### 13.1 Building sustainability *en route* to institutionalization: a tentative hypothesis

Ensuring that innovation adoption is sustained over the long term is a necessary condition to achieve institutionalization. The findings of the present study converge with Frambach and Schillewaert (2002), who suggested that individual adoption depended upon various factors including: attitude toward the innovation (which is similar to faculty perceptions and beliefs in the present study) and organizational facilitators such as training (which is similar to professional development and logistical support), social persuasion (which is similar to the process that is required to create and obtain the approval of institutional plans), and social usage (which could be broadly related to committee involvement, provided that early adopters and seasoned champions participate).

However, Frambach and Schillewaert (2002) noted one area of potential importance, that we did not explore: the role of peer influences on an individual's decision for innovation adoption. Across the life cycle of an institutional change initiative, one can reasonably expect there to be a growing constituency of adopters during the implementation phase, who in-turn, become influencers of potential adopters through the sharing of their ideas and experiences. Considering our findings regarding the importance of individual perceptions and beliefs, we hypothesize that social learning to be the mechanism by which peers can influence each other as suggested by Bui (2015). This approach encapsulated by the cognitive-institutional theory of diffusion, suggests that prospective adopters will obtain the necessary adoption information, informed by an organizational rationale (e.g., Armenakis et al., 1993; Armenakis and Harris, 2002; Haque et al., 2016) to adopt a given innovation. That is, organization will develop the “know-what, know-why, know how” of adopting a given innovation. Thought leaders, associations, practitioners, or influential academics assist in generating collective frames, interpretive schemes (e.g., institutional plans, a common language framework), or prototypes for the organization. Such information provides a lens for individual prospective adopters. Once prospective adopters cross an information threshold (i.e., overcome skepticism and concerns about the innovation) they are more likely to adopt the innovation. Social learning – the underlying mechanism, is manifested through identification with influential practitioners or “champions”, that is, role models who lead by example. Practices such as these are hypothesized to influence future adopters as well as enhancing the odds of attaining normative and/or cognitive-cultural institutionalization.

### 13.2 Implications and practical considerations: increasing the odds for innovation adoption

With a view to create the conditions that optimize the adoption of teaching innovation, below are recommendations that stem from the current study.

#### 13.2.1 Institutional culture: the role of leadership in setting the stage for innovation adoption

To enhance the odds of innovation adoption by faculty, it is recommended that leadership:

• Encourage faculty awareness and understanding of institutional plans and encourage faculty participation amongst potential and seasoned adopters in planning and decision-making committees.
• Foster a sense of self-efficacy and enhanced competence amongst faculty by ensuring they have access to professional development. Furthermore, ensuring access to logistical support (e.g., experiential learning coordinators for experiential learning, and technical support for e-learning) may address concerns about workload (See Professional Dimensions, below).

#### 13.2.2 Perceptions and beliefs

Consistent with the notion that a positive faculty perspective is necessary for innovation adoption, it is essential that leadership:

• Have a communication strategy to show that the proposed innovation is effective for student learning. Showcase faculty who have effectively implemented the teaching innovation, along with student testimonials. Faculty adopters can be platformed through recognition events or through short 2-minute videos to be presented online or as part of professional development workshops.
• Convey the availability of support resources such as professional development workshops, online resources, and logistic support, to demonstrate that the teaching innovation is indeed possible to execute.
• Encourage the formation of teaching networks, so that faculty can seek like-minded colleagues who are engaged in such work. Build faculty's sense of competence and self-efficacy, and confidence by highlighting good teaching practice during the implementation phase amongst individuals in the network.

#### 13.2.3 Professional dimensions and the role of funding

Workload was perceived to be a barrier amongst faculty contemplating the adoption of e-learning. A lack of tradition within the field was negatively associated with the adoption of community-focused experiential learning within teaching practice. Finally, funding was not significantly associated with innovation adoption. As such, these concerns suggest that leadership should:

• Put in policies that reduce workload/time: For example, specially trained teaching assistants or graduate assistants that can reduce or distribute the workload, in both the development and the delivery of the course. Perhaps tie such a policy as part of a funding package.
• Locate and showcase successful examples of community-focused experiential learning by discipline. (This may require examples from other institutions). Frame experiential learning so that it is consistent/compatible with a given discipline. Profile faculty who are engaged in such work, so that they become visible examples for their colleagues.
• Utilize funding for its symbolic value (Jenkins et al., 1998), that is, as a means to highlight, validate, and legitimize the teaching innovation. Funding can be used as a vehicle to invite faculty proposals that advance the teaching innovation. Ensure that faculty who receive funding are profiled or provided a platform, so that their efforts are recognized, and others become aware of this work.

### 13.3 Strengths, Weaknesses and Future Considerations

This study not only affirms the importance of previously known facilitators and barriers for the adoption of e-learning, or community-focused experiential learning, it also assesses their relative importance in terms of their association with adoption behavior. These findings are robust in the sense that we demonstrated the importance of institutional culture variables^13^ as well as faculty perceptions and beliefs across two different forms of teaching innovation. Furthermore, the findings can be explained and understood within the context of established theories of organizational development and innovation adoption. One surprising result was that funding was not significantly associated with innovation adoption, despite the fact that the literature advocates for the availability of funding to enhance faculty adoption (Cook et al., 2009; Glass et al., 2011; Holland, 1999; Newton, 2003; Ward, 1998). However, we urge the reader to treat this finding with caution for at least two reasons: First, given the novelty of this result we would encourage others to seek to replicate these findings. Second, due to limitations in the availability of cases we could not meaningfully assess funding as a range of values. Rather, we treated funding as a binary variable; that is, whether or not faculty received funding. Indeed, except for a few cases, most recipients received about the same amount of funding. (Larger amounts were distributed to the Faculty, rather than the individual).^14^ As such, we could not test whether greater amounts of funding could be associated with adoption behavior. That being said, we were able to explain the non-significant effect of funding by making reference to the behavioral economics literature, which points out the hidden costs of extrinsic motivation (Gallus and Frey, 2016; Kamenica, 2012).

An important limitation of this study was that we likely overestimated the number of adopters, relative to the number of non-adopters. We could see evidence of oversampling when we examined the data comparing adopters versus non-adopters for both the case of e-learning and community-focused experiential learning: In both cases, we had nearly equal numbers of adopters versus non-adopters (See Demographic Characteristics for both e-learning and community-focused experiential learning in the Results section). Clearly this is not the case at our institution. Despite this pattern, the sample was indeed proportional to official counts of gender and proportional to the number of faculty within each of the 10 Faculties at the university. One remedy to mitigate this issue would be to determine the base-rate of the actual number of faculty who are offering e-learning or community-focused experiential learning courses, in a manner that does not rely on faculty responses to a survey. Unfortunately, at the time this study was conducted, there was no institutional mechanism to count courses in an automated fashion. Such information would have enabled us to determine the extent to which oversampling occurred, and to survey more non-adopters to correct for the proportions. Despite these concerns, the results largely converged with that in the extant literature, which gives credence to the validity of the findings.

A second concern was that we had a differential response rate from tenured versus non-tenured faculty, resulting in a sample that had an over-representation of tenured faculty (though we had roughly equal numbers of adopters versus non-adopters across tenured faculty and non-tenured faculty). As such, we have urged the reader to treat the results pertaining to tenure status and its association with e-learning with caution, and we suggest that such a pattern of findings would require additional examination and replication. Furthermore, the combination of low response rate from non-tenured faculty, and likely oversampling of adopters would make it appear that a greater proportion of non-tenured faculty would be adopters. However, note that the tenure status effect only appeared for e-learning, and not for community-focused experiential learning. It leaves open the question why the tenure effect appears in e-learning, but not community-focused experiential learning.

Future research should further examine the construct of faculty perceptions and beliefs, given the fact that only three questionnaire items explained a sizeable amount of variance in the adoption of teaching innovation. Indeed, past research highlights the importance of a positive attitude for members of an organization, toward change. The out-sized effect of faculty perceptions and beliefs bears further scrutiny. Furthermore, to understand how institutionalization occurs, future research should focus upon the role of peer influences in faculty adoption behavior. Finally, the various types of motivation as outlined by Stupnisky et al. (2018) should be further examined in the context of adoption behavior throughout the various phases of organizational change.

## Declarations

### Author contribution statement

Robindra Sidhu: Conceived and designed the experiments; Performed the experiments; Analyzed and interpreted the data; Contributed reagents, materials, analysis tools or data; Wrote the paper.

William H. Gage: Conceived and designed the experiments; Contributed reagents, materials, analysis tools or data; Wrote the paper.

### Funding statement

This research did not receive any specific grant from funding agencies in the public, commercial, or not-for-profit sectors.

### Data availability statement

The data that has been used is confidential.

### Declaration of interests statement

The authors declare no conflict of interest.

### Additional information

No additional information is available for this paper.

## Footnotes

1 Note that for these purposes we are including the literature on the adoption of service learning, community-based research, and community engaged scholarship.

2 Our search examined aspects of teaching where leadership could potentially make a difference within the professional realm. Thus, whether the teaching approach was viewed to be appropriate, and whether faculty could carry it out became the perceptions and beliefs, and self-efficacy measures, respectively. Furthermore, given survey-length constrains, affective factors such as personal interest and enjoyment were not included. That being said, we do not deny the importance of such factors as revealed in our own qualitative research on the adoption of community focused experiential learning (See Lenton et al., 2014).

3 We also asked respondents to indicate the total of the number of credits that they taught in the past five years, for courses that incorporated either e-learning or community focused experiential learning.

4 See https://ethics.gc.ca/eng/policy-politique_tcps2-eptc2_2018.html.

5 Conventionally, a Cronbach's alpha of ≥.70 is considered to be reliable. However, some researchers have questioned the arbitrariness of this cut-off (Cho and Kim, 2015; Taber, 2018). In fact, Cho and Kim (2015) point out that Nunnally (1967) originally stated that a reliability of .5 or .6 was sufficient for exploratory research. By this standard some of our measures fall into this category. Both awareness of institutional plans and degree of logistical support have borderline reliability ranging from .46 to .53. That being said, we believe the measures can be cautiously interpreted at face value, with the caveat that these measures have some degree of measurement error, are exploratory in nature and would benefit with more items, given that the magnitude of alpha varies with the number of items. Also note footnote #8.

6 Contractually limited faculty are full-time faculty whose employment is fixed for a specified period between one to three years and can be extended to five years.

7 There was a total of six individuals who identified as either non-binary, prefer to self-describe, or prefer not to say. We decided to exclude these cases from the logistic regression analyses.

8 The reliability professional development (PD) is clearly below .50 (Table 2), suggesting that the items of the sub-scale were not measuring the same underlying construct (i.e., high measurement error). Upon closer inspection the two items (on campus PD vs off-campus PD) were not correlated (explaining a low KR-20 reliability). This suggests that individuals only chose one source of PD – either on-campus or off. Very few chose both or “other”. Because our primary intent was to determine whether individuals accessed PD, we treated this measure as though we had asked a single question. (i.e., Did you access PD?).

9 In the case of experiential learning, logistical support includes the development of partnership opportunities within the community, upholding risk management policies, partnership agreements and regulations for insurance. In the case of e-Learning, logistical support includes the work of educational developers (in terms of pedagogy) and instructional designers (in terms of technology).

10 For the logistic regression analyses, where betas were negative and the odds ratio was less than one, a reciprocal transformation of the odds ratio was reported so that the results could be described as the odds of not adopting e-learning/experiential learning in the text.

11 We present three measures of pseudo R-square. According to Tabachnick and Fidell (2013), McFadden's R-square provides values that range from 0 to1. However, values of McFadden's R-square are lower than R-square generated from multiple linear regression. In contrast, Cox and Snell pseudo R-square produces values that are higher than McFadden's R-square. However, Cox and Snell pseudo R-square does not range from 0 to 1, rather it has an upper bound less than 1. Nagelkerke R-square represents an adjusted version of Cox and Snell so that values range from 0 to 1. This being said, some authors have suggested that McFadden is the preferred statistic (Allison, 2013). We follow suit.

12 A cursory examination of our dataset shows a general pattern that female faculty are more likely to adopt community-focused experiential learning. We were unable to drill down to the department level, only the Faculty level. Some programs such as Nursing and Social work, are nested within larger Faculties. That being said, these larger Faculties such as Health, and Liberal Arts and Professional Studies (where Social work can be found), did indeed show a greater incidence of female faculty adoption. So, at this stage, it is at best a conjecture that this finding is confounded by discipline.

13 Even though some sub-scales could benefit by utilizing more items, resulting in enhanced reliability.

14 For e-learning, 19 out of 21 respondents reported how much funding they received. The median amount was, $8000, the mean was $25,500 and the range was $2,500 -$75,000. For experiential learning 11 out of 12 respondents indicated how much funding they received. The median amount was $5,000, the mean was $ 10,537. The range was 2000–60,000.

15 Expressed a desire for “better student learning outcomes" pg 3.
